# Building a global early-warning system for environmental risks with remote sensing

**DOI:** 10.1016/j.fmre.2025.11.002

**Published:** 2025-11-17

**Authors:** Libo Wang, Yinglan A, Guoqiang Wang, Baolin Xue, Jin Wu, Yuntao Wang, Qiao Wang

**Affiliations:** a Advanced Interdisciplinary Institute of Satellite Applications, Faculty of Geographical Science, Beijing Normal University, Beijing 100875, China; b College of Water Sciences, Beijing Normal University, Beijing 100875, China

**Keywords:** Environmental risk, Risk early warning, Sky-space-ground integrated monitoring network, Remote sensing, Real-time risk monitoring, Cross-scale observation

## Abstract

Amid intensifying environmental risks, including climate change, biodiversity loss, and pollution, global ecosystems are under unprecedented pressure. Environmental monitoring forms the cornerstone of governance, as ecological risk events unfold through interconnected stages of occurrence, development, migration, and impact. However, current monitoring systems remain fragmented across platforms, regions, and data infrastructures, with inconsistent standards and poor interoperability limiting timely detection and coordinated response. In response to these limitations, we propose a global ecological risk monitoring framework that integrates satellite, aerial, terrestrial, and marine platforms into a hierarchically structured, multi-scale network. This framework supports continuous, cross-scale observation of atmospheric, terrestrial, and aquatic systems, enabling end-to-end lifecycle tracking of ecological and environmental hazards. By synthesizing the strengths of existing monitoring networks and promoting transboundary data interoperability, the system facilitates a transition from passive environmental response to proactive, real-time risk forecasting and decision-making.

## 1 Introduction

Global ecosystems are facing unprecedented challenges due to climate change, biodiversity loss, and pollution, which are becoming increasingly severe. Environmental monitoring is fundamental to effective governance, playing a critical role in detecting, mitigating, and forecasting ecological risks under dynamic environmental conditions. The United Nations Environment Programme (UNEP) emphasized in 2020 that global cooperation and data sharing are essential for effective environmental monitoring, particularly in tracking ecosystem changes and addressing emerging environmental risks [1].

Currently, independent environmental monitoring systems, such as the European Copernicus Programme and the satellite networks operated by the United States, have shown strong capabilities in assessing regional air quality and tracking deforestation [2]. However, these systems still face limitations in capturing the full temporal dynamics of environmental events and assessing their cascading impacts on interconnected systems. In a changing environment, ecological risks and environmental disasters typically follow a sequential process of “occurrence–development–migration–impact,” highlighting the need for integrated monitoring approaches that transcend individual environmental factors, mediums, and regions to comprehensively capture and analyze such events [3].

Despite growing global efforts in environmental monitoring, most national monitoring systems, including those in China, remain uncoordinated. Incompatibilities in standards, technologies, and management frameworks across different networks hinder effective collaboration, making it difficult to achieve global environmental governance objectives [4]. The fragmentation of monitoring systems and the lack of standardized data sharing significantly delay response times and reduce the effectiveness of global efforts to address ecological risks and environmental disasters.

The rapid spread of wildfires, worsening air pollution, and large-scale deforestation provide strong evidence of these challenges, as such events pose severe social hazards and long-term consequences that are difficult to quantify [5]. These discrepancies in monitoring capabilities and data integration represent one of the primary obstacles to implementing global climate policies and conservation initiatives [6]. Therefore, establishing an integrated and interoperable monitoring network is essential for effectively responding to ecological risks and environmental disasters in an increasingly dynamic and uncertain environment.

## 2 Developments and limitations

China’s ecological monitoring efforts began in the 1970s, initially focusing on industrial pollution sources. Over the past decades, the scope has expanded to encompass a wide range of environmental parameters, including air, water, soil, and ecosystems. Today, China has established a multi-tiered monitoring network consisting of national, regional, and local stations [7,8]. While these efforts have played a crucial role in tracking environmental changes, informing policy decisions, and mitigating ecological risks, the current network faces several limitations. Monitoring stations are unevenly distributed, with remote and ecologically fragile areas often underrepresented. Traditional monitoring techniques struggle to keep pace with the detection of emerging pollutants and the rapid dynamics of ecosystem changes, leading to critical gaps in data coverage that limit the system’s ability to comprehensively address environmental challenges. Additionally, fragmented data management frameworks impede cross-sectoral integration and hinder real-time environmental analysis, further constraining the effectiveness of the existing monitoring infrastructure.

Globally, 70% of environmental data remains siloed due to jurisdictional constraints and technical barriers [9]. Many advanced economies, including the United States and the European Union, have developed extensive environmental monitoring systems. However, these systems largely operate independently and lack standardized data formats, limiting their effectiveness in addressing transboundary environmental challenges. These gaps highlight a fundamental global challenge: fragmented monitoring systems are insufficient to tackle transboundary and global-scale environmental risks, emphasizing the urgent need for an integrated and interoperable global monitoring network.

## 3 Way forward

Moving forward, achieving full integration of ecological and environmental monitoring networks will require significant advancements in standardization, technological innovation, and governance frameworks.

For data standardization and harmonization of monitoring methodologies, establishing unified data collection standards and shared frameworks is essential to ensuring interoperability and comparability across global monitoring systems. Seamless integration of these systems will enhance data accessibility and facilitate more effective environmental assessments.

From a technological perspective, advancements in satellite remote sensing and artificial intelligence will enable real-time environmental monitoring, providing more precise predictions and robust decision-making support. These innovations will significantly improve the efficiency and accuracy of environmental monitoring, particularly in addressing pressing global challenges such as climate change, climate-related hydrological responses, and associated ecosystem risks [10].

In terms of governance, establishing a comprehensive global regulatory framework is essential. Countries must strengthen cross-sectoral and transboundary cooperation, developing unified, transparent, and interoperable data management systems. To achieve effective international coordination, we recommend creating a global collaborative platform responsible for standardizing technical protocols and facilitating seamless data exchange across national boundaries. Technological gaps could be addressed through international research partnerships and capacity-building initiatives, while stable funding may be ensured via global funding mechanisms and public-private cooperation. Furthermore, international training programs could help cultivate skilled professionals to sustain the system’s long-term operation.

Through these coordinated actions, an integrated environmental monitoring network can effectively support global ecological governance, enabling evidence-based policymaking aligned with the Sustainable Development Goals (SDGs). In particular, the proposed early-warning system directly contributes to SDG 13 (Climate Action), SDG 14 (Life Below Water), and SDG 15 (Life on Land). By offering timely and accurate ecological risk assessments and actionable alerts, this system enhances proactive environmental management, ultimately strengthening global sustainability efforts.

To effectively respond to major ecological and environmental disasters driven by climate change, an integrated monitoring network must facilitate end-to-end monitoring across the entire disaster lifecycle—encompassing occurrence, development, migration, and impact. The emphasis will be on continuous and multi-scale monitoring across temporal phases, geographic regions, environmental factors, and spatial scales. This will be achieved through the standardization of monitoring protocols, technological integration, and harmonized management frameworks, ensuring seamless and comprehensive disaster tracking throughout all stages of “occurrence–development–migration–impact.”

Building on this integrated monitoring framework, the next critical step lies in advancing risk assessment and early-warning capabilities. By leveraging the continuous data streams generated through multi-scale observations, future efforts should aim to develop dynamic risk profiling systems that detect anomalies, anticipate disruptions, and inform timely responses. This includes designing threshold-based alerts, predictive modeling approaches, and real-time analytical tools that can translate raw monitoring data into actionable warnings for decision-makers.

**Fig. 1:**
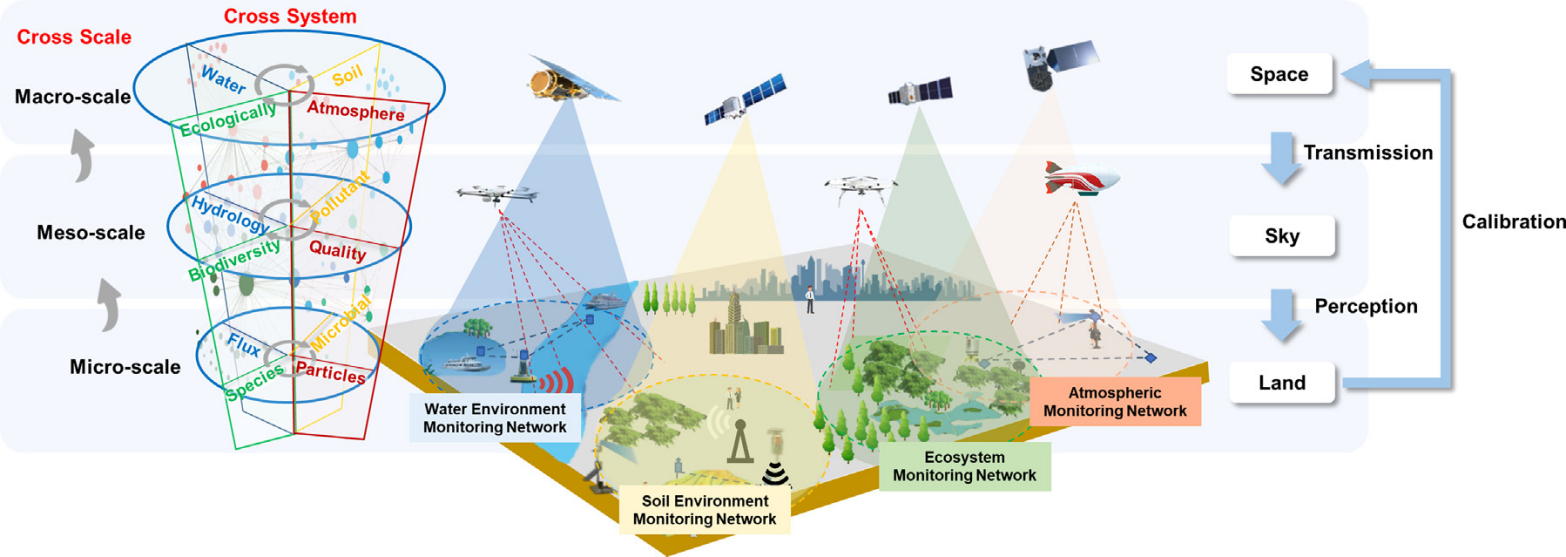
**A conceptual framework for designing an integrated environmental monitoring network**.

To address the diverse monitoring demands across different systems, spatial scales, and environmental factors, a vertically structured monitoring network is proposed, spanning space, the atmosphere, and terrestrial environments (Fig. 1). This multi-scale framework comprises water, soil, ecological, and atmospheric monitoring networks at macro, meso, and micro levels. By ensuring data interoperability and standardizing technical protocols across these networks, heterogeneous monitoring systems can be seamlessly integrated, thereby improving monitoring precision and real-time responsiveness. Ultimately, this approach facilitates the development of a fully interoperable and integrated environmental monitoring network.

Scholars, leveraging comprehensive observational datasets, can reconstruct the full dynamics of disaster events and generate new theoretical insights. Policymakers, equipped with seamlessly integrated monitoring data, will be able to track disaster progression and migration in real time, enabling proactive early warning dissemination and the formulation of coordinated response strategies to minimize impacts. Additionally, businesses can harness the monitoring network’s data infrastructure to enhance public services and improve societal resilience to environmental hazards.

Regarding the practical implementation of an integrated monitoring network, China is set to spearhead major initiatives. With a well-established monitoring framework encompassing environmental domains such as water, soil, atmosphere, and ecosystems—spanning ground-based, space-based, airborne, and ocean-based platforms—China has developed relatively mature standards, technologies, and equipment. By harmonizing and enhancing its existing spaceborne, airborne, ground-based, and oceanic networks, China aims to lay the groundwork for developing an event-driven “sky-air-ground-sea” integrated monitoring system. China will continue advancing the integration of satellite technology with ground-based monitoring infrastructure, serving as a reference model for other countries and contributing to the advancement of global environmental governance and sustainability.

The integration of ecological and environmental monitoring networks is a fundamental prerequisite for achieving global environmental sustainability. As one of the world’s most biodiverse and rapidly urbanizing nations, China’s innovations in environmental monitoring offer a blueprint for other countries facing similar challenges. By harmonizing its strategies with global sustainable development goals, China is well-positioned to play a pivotal role in shaping the future of ecological conservation. With accelerating environmental change, a resilient and adaptive monitoring system is no longer a luxury but a necessity. When environmental monitoring technologies shift from tools of control to instruments of understanding, humanity can truly advance toward ecological harmony. Recognizing this urgency, China is poised to embrace this opportunity, strengthening its environmental monitoring capabilities to drive ecological progress and deliver lasting benefits to both its citizens and the planet.

## CRediT authorship contribution statement

**Libo Wang:** Writing – review & editing, Writing – original draft, Investigation, Formal analysis, Conceptualization. **Yinglan A:** Writing – review & editing, Writing – original draft, Conceptualization. **Guoqiang Wang:** Writing – review & editing, Writing – original draft. **Baolin Xue:** Writing – review & editing. **Jin Wu:** Writing – review & editing. **Yuntao Wang:** Writing – original draft. **Qiao Wang:** Writing – review & editing, Conceptualization.

## Declaration of competing interest

The authors declare that they have no conflicts of interest in this work.
